# Characteristics and perceived suitability of artificial intelligence-driven sports coaches: a pilot study on psychological and perceptual factors

**DOI:** 10.3389/fspor.2025.1548980

**Published:** 2025-05-12

**Authors:** Carlo Dindorf, Jonas Dully, Eva Bartaguiz, Tessa Menges, Claudia Reidick, Johann-Nikolaus Seibert, Michael Fröhlich

**Affiliations:** ^1^ Department of Sports Science, RPTU University of Kaiserslautern-Landau, Kaiserslautern, Germany; ^2^ Endurance Coach GmbH, Berlin, Germany; ^3^ Chemistry Didactics, RPTU University of Kaiserslautern-Landau, Kaiserslautern, Germany

**Keywords:** training support, human-computer interaction, personalized coaching, large language models, coaching styles, artificial intelligence personalities, user engagement, smart coaching

## Abstract

**Introduction:**

Access to human sports coaches is often limited by financial and logistical barriers, leading to disparities in the availability of high-quality coaches. Artificial intelligence (AI) coaches powered by Large Language Models (LLMs) might offer promising means to augment human coaches by supporting or autonomously performing specific coaching tasks within targeted domains. This study investigated AI coaches' associated attributes and perceived suitability in training contexts by addressing three primary questions: (A) Which attributes on a semantic differential scale effectively describe the dimensions of AI coaches in the context of training support? (B) Do participants with varying perceptions of AI suitability for their training practices differ in the attributes they associate with AI coaches, as measured by a semantic differential scale? (C) Do different individual achievement motives (AMS)-Sport influence the perception of AI coaches' suitability?

**Methods:**

The study comprised two parts. The first involves the development of a semantic differential scale to quantify the perceptions of AI coaches and an analysis of how different AI coach personalities, designed using an LLM, are perceived concerning their training suitability and how achievement motives influence these perceptions. Six distinct AI coach personalities were created to reflect the diverse coaching styles.

**Results:**

Factor analysis revealed four key dimensions of AI coach attributes: *knowledge transfer*, *goal-oriented persistence*, *appreciation and recognition*, and *motivational support*. The results indicated that coaches rated as more suitable exhibited supportive traits, such as motivation and goal orientation, compared to those rated less suitable. Participants with a lower Fear of Failure (FoF) also tended to rate AI coaches as more appropriate.

**Conclusion:**

These findings underscore the importance of aligning AI coaches' characteristics with their motivational profiles to improve user engagement.

## 1 Introduction

Coaches are pivotal in guiding individuals to enhance their skills, knowledge, and performance in various domains. Coaches are indispensable for developing athletes' technical abilities, physical conditioning, mental resilience, and strategic understanding of sports. Beyond these competencies, coaches tailor training programs to individual needs and offer mentorship that empowers athletes to achieve their full potential. Research suggests that having a coach can positively affect athletic performance and development. Athletes with coaches demonstrate better task compliance and break-time adherence during training (1). Coached athletes also reported higher personal and social skill development levels than untrained coaches (2). Coaches play a crucial role in developing athletes' mindsets, which can improve their performance in both sports and life (3). However, the accessibility of human coaches can be limited by financial constraints and availability, creating disparities in access to high-quality coaching (4).

Conversational artificial intelligence (AI) models have significant potential for transformative applications in diverse domains (5–7). Using natural language processing (NLP), these systems engage users in meaningful dialogues, analyze data, and deliver customized solutions. Their 24/7 availability and ability to automate routine tasks render them highly accessible and practical. Recent advances in AI-powered chatbots have demonstrated their ability to provide personalized education, healthcare, and customer service guidance. For example, AI language-teaching tools assist learners with language acquisition through interactive exercises and customized feedback, enhancing their educational experiences (8). In healthcare, platforms utilize conversational AI to help users assess symptoms and provide tailored health advice (9). Similarly, customer service applications streamline user interactions by efficiently resolving queries and offering customized recommendations based on user data (10).

AI coaches powered by Large Language Models (LLMs) might offer promising means to augment human coaches by supporting or autonomously performing specific coaching tasks within targeted domains. These systems can act as virtual assistants guiding athletes through workouts, recovery, and mental preparation with data-driven insights. Furthermore, AI systems offer scalable and cost-effective solutions to democratize individualized training and coaching access. Additionally, AI coaches can draw on extensive background knowledge, potentially surpassing less experienced or less theoretically informed human coaches in contexts where theoretical expertise is crucial. Moreover, the younger generation's affinity for digital technologies (11) may enhance their engagement with and receptivity to this coaching approach. However, adopting lifestyle modifications, health promotion, and sports remains limited (12). Commercial applications, such as platforms for endurance training, incorporate LLMs for user coaching (e.g., enduco, endurance coach GmbH, 10245 Berlin, Germany). However, rigorous scientific validation of their effectiveness remains pending. Preliminary studies suggest that AI chatbots can effectively promote physical activity; however, their utility in dietary modification and weight management has shown mixed results (12). Similarly, AI-driven systems in sports coaching are still in their infancy, although early evidence suggests that they can enhance training experiences by analyzing performance data and tailoring individualized programs (13). Case studies have shown that personalized AI-guided training approaches lead to higher engagement, increased motivation, and improved performance outcomes compared to standardized training methods (13, 14).

Human-computer interaction (HCI) frameworks highlight the importance of usability, engagement, and trust in the successful design and adoption of AI tools (15, 16). A key challenge in integrating AI into sports coaching is understanding how users perceive these systems. Effective measurement tools are essential to capture these perceptions. Semantic differential (17) provides a structured method for evaluating attributes by capturing individuals' attitudes toward a specific concept through bipolar adjectives. Semantic differentials have been utilized in sports-related contexts, such as analyzing everyday perceptions of the term “training” (18) or examining the meaning of a sports event experience among active sports tourists (19). In the context of AI coaches, semantic differentiation enables researchers to identify how athletes perceive and evaluate various attributes of these systems, such as competence and motivational effectiveness. This is particularly relevant because studies have suggested that chatbots that provide motivational and social support are more likely to sustain user engagement and adherence to training practices (20, 21). However, to the best of our knowledge, no existing tools specifically evaluate the perceptions of AI coaches in the context of sports training (A).

An immediate application of such a semantic differential involves analyzing how AI coaches with varying levels of perceived suitability for the user's own training practices are linked with specific attributes. Although general research has highlighted the traits of effective human sports coaches (22), little is known about how athletes perceive the suitability of AI coaches for their training practices, mainly through a semantic differential framework (B). In this context, suitability refers to how effectively a coach is perceived in supporting a trainee's individual training goals and needs. Understanding these associations can offer insights into which end users most value characteristics and how AI coaches can be refined to meet user needs better. Findings in AI applications in sports (23) indicate that technology readiness and perceived usefulness influence users' willingness to use AI Sports, suggesting that similar factors may play a role in sports coaching.

Self-determination theory (24) posits that motivation and well-being are strongly influenced by fulfilling three basic psychological needs: competence, autonomy, and relatedness. Research has underscored that satisfying these needs is essential for fostering sustained engagement and commitment to sports activities, thereby increasing the likelihood of long-term physical activity among athletes (25, 26). Within the coaching context (27), highlight coaches' autonomy-supportive behaviors can significantly enhance athletes' intrinsic motivation and overall well-being in their motivational model. This highlights the pivotal role of the coach-athlete relationship, which is inherently shaped by motivational dynamics.

A concrete example of these dynamics may also be observed in athletes' achievement motivation (28), which is characterized by two dimensions: Hope for Success (HfS) and Fear of Failure (FoF). These dimensions may also influence athletes' trust, engagement, and acceptance of AI coaching systems. Research has indicated a significant relationship between coaching style and athletes' FoF and HfS scores. Studies have shown that controlling coaching behaviors are linked to heightened FoF, whereas autonomy-supportive styles are related to reduced FoF (29). High-quality coach-athlete relationships, marked by closeness, commitment, and empathy, can predict and reduce FoF (30).

In the context of AI coaching, athletes with high HfS may view AI systems as valuable tools for enhancing performance through data-driven feedback. In contrast, those with a high FoF may express concerns about exposing weaknesses or lacking emotional support. Therefore, understanding these motivational factors is crucial for designing AI coaching systems that cater to athletes' diverse needs. However, to the best of our knowledge, no empirical studies have explored these dynamics within the realm of AI coaching (C).

In summary, despite growing interest in AI coaching, existing research lacks validated tools to assess user perceptions and fails to account for how individual differences in achievement motives influence acceptance. Addressing these gaps is crucial for advancing theoretical frameworks in human-AI interaction, enhancing user-centered design, and determining whether AI-driven coaching can serve as viable alternatives or complementary tools for human sports coaches in specific coaching tasks. Consequently, with the derived research deficits (A), (B), and (C), this study aims to investigate the following overarching research questions, utilizing data from two separate data collections:

• (R1) Which attributes on a semantic differential scale effectively describe the dimensions of AI coaches in the context of training support? (Study 1 & 2)
• (R2) Do coaches with varying perceptions of AI's suitability for their training practices differ in the attributes they associate with AI coaches, as measured by a semantic differential scale? (Study 2)
• (R3) Do different individual achievement motives influence the perception of AI coaches' suitability? (Study 2)

This study seeks to deepen the understanding of the interplay between motivational factors and acceptance of AI coaching systems. This study aims to provide actionable insights for developing AI coaches tailored to athletes' needs by identifying the key characteristics and attributes contributing to perceived effectiveness.

## 2 Method

### 2.1 Development of AI coaching systems

Six distinct AI-based coach personalities were included in the study. AI coaches were developed using GPT-4 (OpenAI, San Francisco, CA, USA), accessed via OpenAI's API, to integrate the model into the system. Table 1 provides an overview of their intended characteristics as conceptualized by the development team. While this summary reflects design intentions, individual perceptions of these coaches may vary, as explored later in the study. The coaches were designed to emulate specific types of real-life coaches, each characterized by their unique expertise, motivational styles, and communication approaches. Their primary purpose was to engage users in concise, training-focused conversations to increase their motivation for upcoming sessions. By maintaining a brief yet supportive communication style, coaches strive to inspire, guide, and help users stay focused on their next training goals.

**Table 1:** Description of the different artificial intelligence coach personalities used.

Coach name	Attempt to describe its properties
C_MindfulMotivator	A mindful coach and former elite cyclist who aims to empower athletes with positive affirmation and visualization to reach peak mental and physical performance
C_GoalDrivenAnalyst	A former professional cyclist who aims to motivate the athlete based on the big picture of training phases and the goal they want to achieve and evaluates the training based on the data from sessions
C_HumorousEncourager	A former top runner who aims to motivate the athlete with humor and throws jokes to loosen up the mood
C_DisciplineDriver	An ex-military who turned into an endurance coach. Pushes the motivation through drills and discipline with a firm tone
C_ReflectiveHumorist	A reflected cycling coach who aims to find the right words for his athletes and uses humor for motivation
C_MindfulGuide	A mindfulness coach and cycling and running instructor who aims to help athletes find mental balance for improved performance with a calm and relaxing tone

Each person was defined by a specific prompt to model distinct personalities with tailored motivational and communicative strategies. The conceptualization of the six coach personas was grounded in aspects from psychology, motivational interviewing, and cognitive-behavioral frameworks:

• **C_MindfulMotivator** and **C_MindfulGuide** were inspired by mindfulness-based interventions, which have been shown to enhance mental focus and performance (31).
• **C_GoalDrivenAnalyst** draws upon goal-setting theory and data-driven training approaches, underscoring the role of structured planning and measurable objectives in athletic performance (32).
• **C_HumorousEncourager** and **C_ReflectiveHumorist** were informed by research on the role of humor in motivation and stress reduction in sports contexts (33).
• **C_DisciplineDriver** was based on principles observed in high-performance and military training environments, where discipline, structured feedback, and authoritative communication are critical (34).

For each persona, explicit linguistic style guidelines were developed to ensure that the language used by the coaches accurately reflected their intended motivational strategies and expert backgrounds. Coaches emphasizing empathy and reflective support (e.g., C_MindfulMotivator and C_MindfulGuide) utilized affirming language, reflective statements, and a calm tone. Coaches with a focus on goal setting and performance analysis (e.g., C_GoalDrivenAnalyst) employed precise, data-informed language with an emphasis on structured feedback. The humor-based personas (C_HumorousEncourager and C_ReflectiveHumorist) integrated situational humor that was carefully balanced to maintain motivational intent without detracting from the training focus. The authoritative and disciplined approach of C_DisciplineDriver was achieved through concise, directive language that mirrors the communication style typical of military training.

The coaches were designed to integrate into an app facilitating endurance training planning. The prompt structure was as follows:

• Coach Role: The prompt included a brief description of the app, specifying that the persona acts as a human-like coach within the app, supporting users in all aspects of their training.
• Persona Description: Each coach persona is assigned a name, sport, key personality trait, or illustration to create the impression of interacting with a real person.
• Communication Focus: The prompt restricted communication to training-related topics only, explicitly preventing the discussion of unrelated subjects.
• Text Style: Personas use concise text and incorporate emojis to maintain engagement and a friendly tone.
• No External References: The prompt ensures no quotes from the critical app information uploaded into the GPT, from which the personas draw factual content.
• Data Format: Interactions are structured in JSON format for efficient data handling.

The development of the coach personas followed an iterative design process. Initial prompt drafts were created based on the aforementioned foundations and linguistic guidelines. These drafts underwent multiple rounds of review and refinement, including an expert evaluation by professionals in sports science and training practice to assess whether the intended motivational styles were distinct and aligned with academic principles. This was followed by a pilot testing with representative user groups to gather feedback on the perceived distinctiveness and motivational impact of each persona. Adjustments were made based on this feedback.

To ensure standardized evaluation and minimize confounding variables, participants were presented with pre-extracted chat logs rather than engaging in real-time interactions with the AI coaches. This approach allowed for strict control over the conversational content, ensuring that all participants evaluated the coaches based on identical scenarios. While this design does not capture the dynamic adaptability of live AI interactions it was essential for isolating perceptual differences between coaches, free from variability in user input or system responsiveness. The selected scenario (a user reporting underperforming sessions and seeking motivation) was designed to reflect a common coaching use case, with full transcripts provided in the Supplementary Material for transparency. The names of the AI coaches were concealed from participants to avoid bias.

### 2.2 Overview of the empirical study workflow

**Figure 1:**
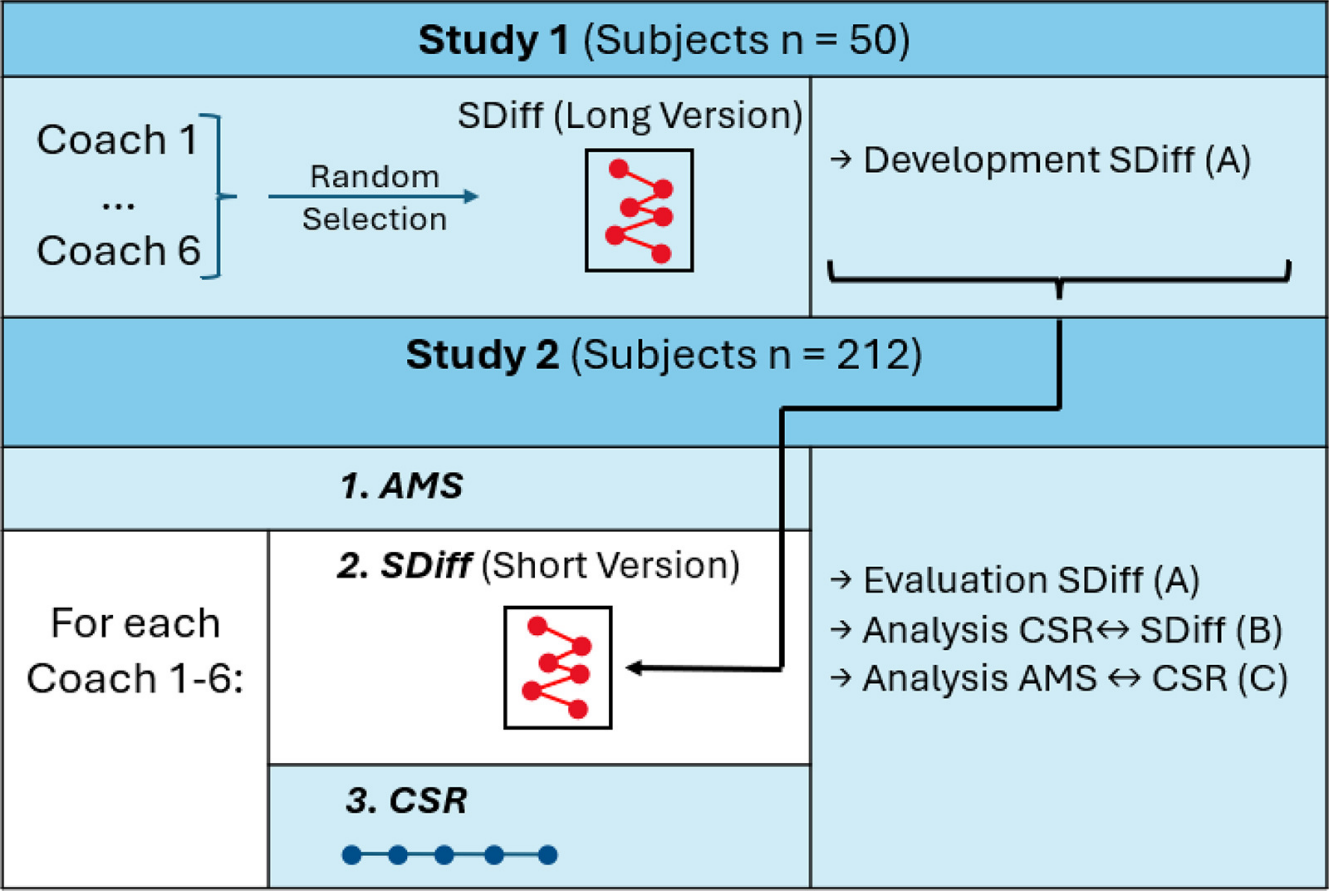
Workflow overview (SDiff, semantic differential; AMS, individual achievement motives sport questionnaire; CSR, coach suitability rating).

This study was divided into two phases: Study 1 and Study 2 (see Figure 1 for a visual overview). Study 1 focuses on the development of a semantic differential. This phase involved creating an extensive version of the semantic differential by identifying potentially relevant attribute pairs. Participants rated a randomly selected chat transcript from one of the six AI coaches using a comprehensive version of the semantic differential. Subsequently, the semantic differential is refined by identifying the underlying dimensions and removing specific attributes. Study 2 builds on the findings of Study 1. It began with participants completing the achievement motives (AMS)-Sport questionnaire, followed by exposure to chat transcripts from six AI coaches, presented in randomized order. The participants then evaluated the suitability of each AI coach for personal training using the semantic differential developed in Study 1. The methodology for these steps is described in detail in the following section.

### 2.3 Participants and data acquisition

All the participants received detailed information about the study and provided informed consent. Participation was restricted to legal adult individuals (18 years or older) actively engaged in sports. The study adhered to the principles of the Declaration of Helsinki and ensured that all ethical considerations were addressed. The studies were approved by the Institutional Ethics Committee of University of Kaiserslautern-Landau. Both studies conducted online surveys using LimeSurvey (LimeSurvey GmbH, Hamburg, Germany). The survey in Study 1 required approximately 7 min to complete, whereas the survey in Study 2 took approximately 30 min. Participants were recruited through the digital distribution of a survey link shared within German sports clubs to ensure they were actively engaged in sports. Study 1 adopted an exploratory approach using EFA to identify the potential factors subsequently evaluated. In this context, a sample size of 50 can be considered a reasonable minimum for exploratory research (35). For the subsequent CFA, based on the moderate complexity of the identified factor structure, a sample size of 212 participants was collected. This exceeds the minimum recommendation of 200 participants, particularly for models of moderate complexity, outlined by (36) and (37). Participant characteristics for both studies are summarized in Table 2.

**Table 2:** Participants characteristics of the two studies.

Characteristic	Study 1	Study 2
*N* participants	50 21 men, 29 women, 0 divers	212 95 men, 117 women, 0 divers
Age	25.63 ± 8.56 years	26.81 ± 7.77 years
Weekly hourly physical activity	7.30 ± 3.88 h	8.02 ± 5.31 h

### 2.4 Measurement instruments

#### 2.4.1 Semantic differential

The development of semantic differentials began by exploring the attributes commonly associated with sports coaches. We conducted a workshop with 30 voluntary sports students to gather insights, asking them to identify their associated attributes with sports coaches. While experienced coaches might offer deeper insights from the sports practice, we prioritized students for three reasons: as primary potential users of AI coaching tools, their perceptions directly reflect target audience needs [consistent with user-centered design approaches in sports technology; see (38)]; participants represented diverse sports disciplines, providing broader attribute coverage than sport-specific coaches might; less experienced trainees often articulate coaching principles more explicitly, whereas experts may rely on internalized, tacit knowledge developed through prolonged practice (39).

The mentioned attributes were recorded on a shared dashboard that was visible to all the participants and was refined collaboratively until no additional attributes were suggested. Building on this initial list, the participants were tasked with consolidating similar attributes by merging them with overlapping meanings, retaining only the most relevant and easily understood. In case of disagreement, potentially conflicting attributes were retained. Subsequently, two sports scientists independently created attribute pairs for a semantic differential to ensure each pair included one attribute and its opposite. Discrepancies between the two experts were resolved collaboratively, leading to a final set of attribute pairs—this initial version of the semantic differential comprised 24 items.

In the online survey for Study 1, the participants were asked to evaluate a randomly selected coach chat using this semantic differential with all identified attribute pairs. An exploratory factor analysis (EFA) was conducted to understand the underlying structure of the attributes and enhance the usability of the semantic differential for subsequent analysis. This analysis aimed to identify the core dimensions of coach ratings, assess the relationships between items, and streamline the semantic differential for greater efficiency. Principal component analysis with varimax rotation was employed, guided by eigenvalues exceeding 1.0, scree plots, and content validation. Items with ambiguous loadings or involvement in multiple factors were systematically excluded following expert assessments and iterative refinement of the factor analysis.

To examine the robustness and generalizability of the derived factor structure, we conducted confirmatory factor analysis (CFA) using the independent sample from Study 2. The CFA aimed to determine whether the factor solutions identified in previous exploratory analyses could be replicated in this sample. By conducting CFA on a separate sample, we validated the factor structure, providing evidence of the stability of the factors across different datasets and ensuring that they consistently measured the intended constructs.

Owing to the multiple measurements per subject in Study 2, a single randomly selected coach result per subject was selected to meet the requirements of the CFA. The model was estimated using Maximum Likelihood estimation. The fit of the hypothesized model was evaluated using various fit indices, and factor loadings were standardized to assess the strength of the relationships between the observed variables and their respective latent factors. To further evaluate the reliability of the latent factors, we calculated Cronbach's alpha.

After identifying each factor, the original item values were aggregated using the mean. In particular, we considered the loading direction to derive a comprehensive factor value for subsequent calculations. For all applied semantic differentials, attributes were presented in a randomized order to minimize positional bias, including the absolute positioning of items and the sequence of paired attributes.

#### 2.4.2 Coaches perceived suitability

Building on (40), which uses a Likert scale to measure the acceptance of technologies, this study also employed a five-point Likert scale (1 = strongly disagree, 5 = strongly agree) to assess the perceived suitability of the AI coaches for individual training. After each semantic differential, participants were asked the following question (originally in German): “To what extent does the following statement apply to you: ‘I would want to use this coach for supporting my training.’” This question was intentionally framed to gauge each AI coach's perceived relevance and personal appeal, thus directly measuring their potential for real-world applications.

#### 2.4.3 AMS-Sport questionnaire

The Achievement Motives Scale-Sport (AMS-Sport), which measures two components of achievement motivation, HfS, and FoF, was used to assess motivational dynamics relevant to athletic performance and their influence on behavioral outcomes (28, 41). While AMS-Sport allows for calculating aggregate measures such as net hope and overall achievement motivation, combining the two components, HfS and FoF, into a single metric can obscure the crucial distinctions between these two motivational constructs. Research has consistently shown that HfS and FoF represent distinct psychological mechanisms that influence behaviour and performance differently. Specifically, HfS is associated with approach motivation, in which individuals are driven by the desire to achieve success. In contrast, FoF is linked to avoidance motivation, in which individuals primarily focus on preventing failure (42). Combining these components into a single metric compromises the clarity of motivational profiles and limits our ability to understand the complex motivational dynamics at play fully. Therefore, this study separately examines HfS and FoF to preserve the distinction between approach and avoidance motivations and enhance the interpretability of motivational outcomes.

The AMS-Sport is available in an extended version, with 15 items per component, and a short version, with five items per component. For this study, the short version of the AMS-Sport (43) was selected for its efficiency and economic use. After calculating the motive values for HfS and FoF, the data were grouped into three approximately equal-sized categories (low, medium, and high) per motive based on percentiles. This grouping was performed as a preliminary step in further analyses.

### 2.5 Statistics and further calculations

The EFA was performed using SPSS software (IBM, version 29; SPSS Inc., Chicago, IL, USA). We used the lavaan package (44) in R to perform CFA. Cronbach's alpha was calculated using the psych library (45) in R. The Friedman tests were calculated for each dimension to compare the different coaches' effect sizes calculated with Kendall's W. The Friedman test was chosen because the variables are on an ordinal scale, and the different coaches can be seen as repeated-measures factors since the questionnaires are the same (46).

To evaluate the effects of FoF, HfS, and coaches on perceived suitability*,* FoF and HfS were seen as between-subject effects, and coaches were seen as a repeated measures effect (within-subject effects). Because the data were non-normally distributed, a non-parametric F2-LD-F1 design with Wald-type statistics was used with the R (47) package *nparLD* (48)*.* Significant main and interaction effects were compared *post-hoc* using non-parametric procedures in R. For group effects (FoF and HfS), Kruskal–Wallis tests with Bonferroni corrections were used, followed by Dunn's tests for significant results. The effect sizes r and d for Dunn's tests were calculated by dividing the test statistic by the square root of the sum of the group sizes (49). A Friedman Test was conducted for the effect of coach, which was seen as a time-dependent factor, followed by exact pairwise tests with Bonferroni correction using the *PMCMRplus package* (50). The effect sizes for the Friedman tests were calculated with Kendall's coefficient of concordance (Kendall's W) value by dividing the test statistic (*χ*^2^) by the product of several participants (*N*) and the times of measurements minus 1 (K−1) (51). Evaluating differences of *FoF* inside each coach, Kruskal–Wallis tests were performed with effect sizes Eta-Squared (*η*^2^) (49).

Based on (52) and (53), the rating scale used was assumed to be interval-scaled for the visual presentation of the results. Therefore, the bootstrap mean and 95% confidence interval were calculated using 1,000 bootstrap samples. Visualization was performed in Python using the Seaborn library (54).

## 3 Results

### 3.1 Semantic differential results

The results of the exploratory factor analysis showed that both the Bartlett test [chi-square (45) = 227.23, *p* < 0.001] and the Kaiser–Meyer–Olkin measure of sampling adequacy (KMO = 0.74) indicated that the variables were suitable for factor analysis. Eigenvalues greater than 1.0, scree plots, and content validation suggested a four-factor solution explaining 79.36% of the variance. Owing to ambiguous loading and/or the involvement of multiple factors following expert assessments, the following items were excluded from the factor solution: helpful | unhelpful; positive | negative; precise | imprecise; interesting | uninteresting; empowering | disempowering; confidence-boosting | confidence-decreasing; supportive | hindering; strengthening | weakening; close | distant; mood-lifting | mood-decreasing; encouraging | discouraging; humorous | serious; relaxed | strict; and patient | impatient.

CFA confirmed the factor structure identified in the EFA, demonstrating a strong and well-fitting model. Fit indices indicated excellent model fit: the Chi-square statistic was 48.45 (df = 29, *p* = 0.01), which, while significant, suggests a good fit given the model's complexity (55). Additional fit indices included a Comparative Fit Index (CFI) of 0.98 and a Tucker–Lewis index (TLI) of 0.97, both above the acceptable threshold of 0.90 (56), further supporting a good model fit. The Root Mean Square Error of Approximation (RMSEA) was 0.06, within the acceptable range (≤0.08) (57), and the Standardized Root Mean Square Residual (SRMR) was 0.04, well below the recommended threshold of 0.08 (56), indicating minimal residuals and an adequate fit.

Factor loadings for all observed variables were substantial and statistically significant (*p* < 0.001), ranging from 0.76 to 0.93 (see Table 3 for the standardized factor loadings). These results and Cronbach's alpha values of 0.88, 0.86, 0.86, and 0.90 for Factors 1–4 confirmed high internal consistency. Furthermore, significant positive covariances between the latent factors, such as the covariance between F3 and F4 (estimate = 0.87, *p* < 0.001), suggest that the latent constructs are interrelated.

**Table 3:** Standardized factor loadings from the confirmatory factor analysis are presented, with the attributes displayed alongside their original formulations in German.

Item	Factor 1	Factor 2	Factor 3	Factor 4
informative | contentless wissensvermittelnd | inhaltslos	.806			
insightful | confusing aufschlussreich | verwirrend	.872			
educational | not educational lehrreich | nicht-lehrreich	.847			
persistent | yielding hartnäckig | nachgiebig		.767		
determined | indecisive entschlossen | unentschlossen		.874		
goal-oriented | aimless zielorientiert | ziellos		.827		
appreciative | disregarding anerkennend | missachtend			.887	
rewarding | punishing belohnend | bestrafend			.855	
energizing | exhausting nergiegebend | entkräftend				.896
motivating | demotivating motivierend | demotivierend				.930

According to the mean bootstrap values, the lowest suitability rating was observed for the C_HumorousEncourager (2.15 ± 0.09). The coaches progressively become more suitable, with C_ReflectiveHumorist (3.15 ± 0.07), followed by C_DisciplineDriver (3.34 ± 0.08), C_MindfulMotivator (3.51 ± 0.07), C_MindfulGuide (3.57 ± 0.07), and, ultimately, C_GoalDrivenAnalyst (3.68 ± 0.07) being regarded as the most suitable.

**Figure 2:**
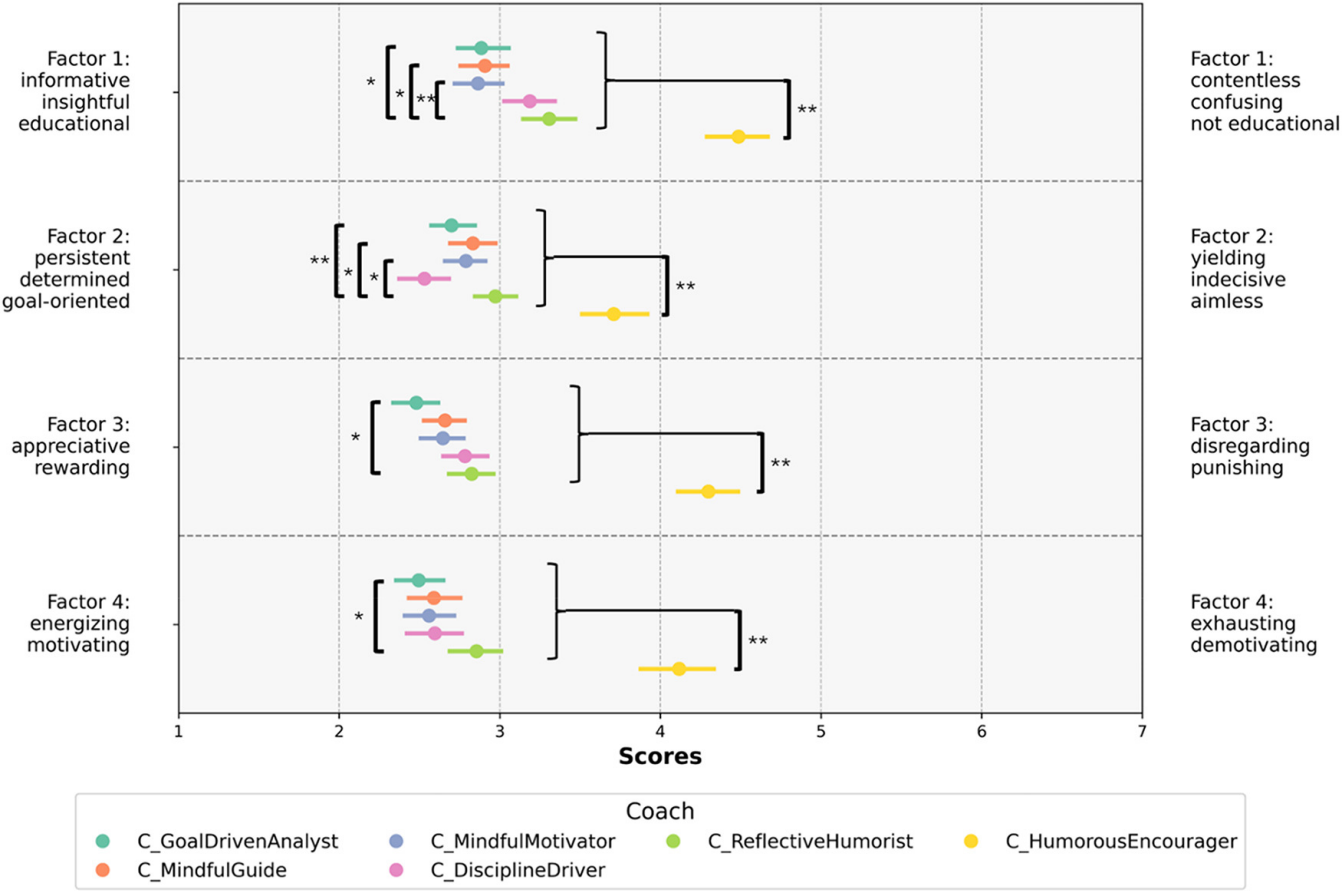
Bootstrap (*n* = 1,000) means and 95% confidence intervals of the factor scores for each coach separately. The coaches are ordered according to their bootstrap mean ranking regarding suitability for the participants’ training practice (see next section). *p < 0.05, **p < 0.001.

Based on the factor solution identified, according to non-parametric rank-based statistics, there are significant effects for coach for every factor in the semantic differential (F1: *χ*^2^(5, *n* = 198) = 172.098; *p* < 0.001; F2: *χ*^2^(5, *n* = 196) = 169.81; *p* < 0.001; F3: *χ*^2^(5, *n* = 198) = 190.18; *p* < 0.001; F4: *χ*^2^(5, *n* = 198) = 127.07; *p* < 0.001). Figure 2 shows the factor values for each coach. The corresponding *post hoc* test results are presented in the figure.

Humorous Encourager, who received the lowest rating regarding suitability for the participants' training practices, demonstrated a shift across all factors in the semantic differential to attribute dimensions representing contentless, yielding, disregarding, and exhausting attributes. Conversely, C_GoalDrivenAnalyst, rated as the most promising for participants' training practices, demonstrated a significant shift toward opposite attribute dimensions, representing informative, persistent, appreciative, and energizing attributes. In addition to the pronounced differences observed with the C_HumorousEncourager across all factors, additional distinctions were apparent among the other AI coaches in the factors examined.

### 3.2 Perceived suitability for own training

#### 3.2.1 Global test

According to non-parametric Wald statistics, comparing the perceived suitability of the coaches, there is a significant interaction effect of the coaches and FoF groups [Wald *χ*^2^(10) = 19.254, *p* = 0.037]. No interaction between the coaches and the groups of HfS [Wald *χ*^2^(10) = 8.111, *p* = 0.618] and the coaches and both FoF and HfS [Wald *χ*^2^(20) = 17.30, *p* = 0.633] are found. There is also no main effect for HfS [Wald *χ*^2^(2) = 0.015, *p* = 0.993].

#### 3.2.2 Coach difference inside each group of FoF

**Figure 3:**
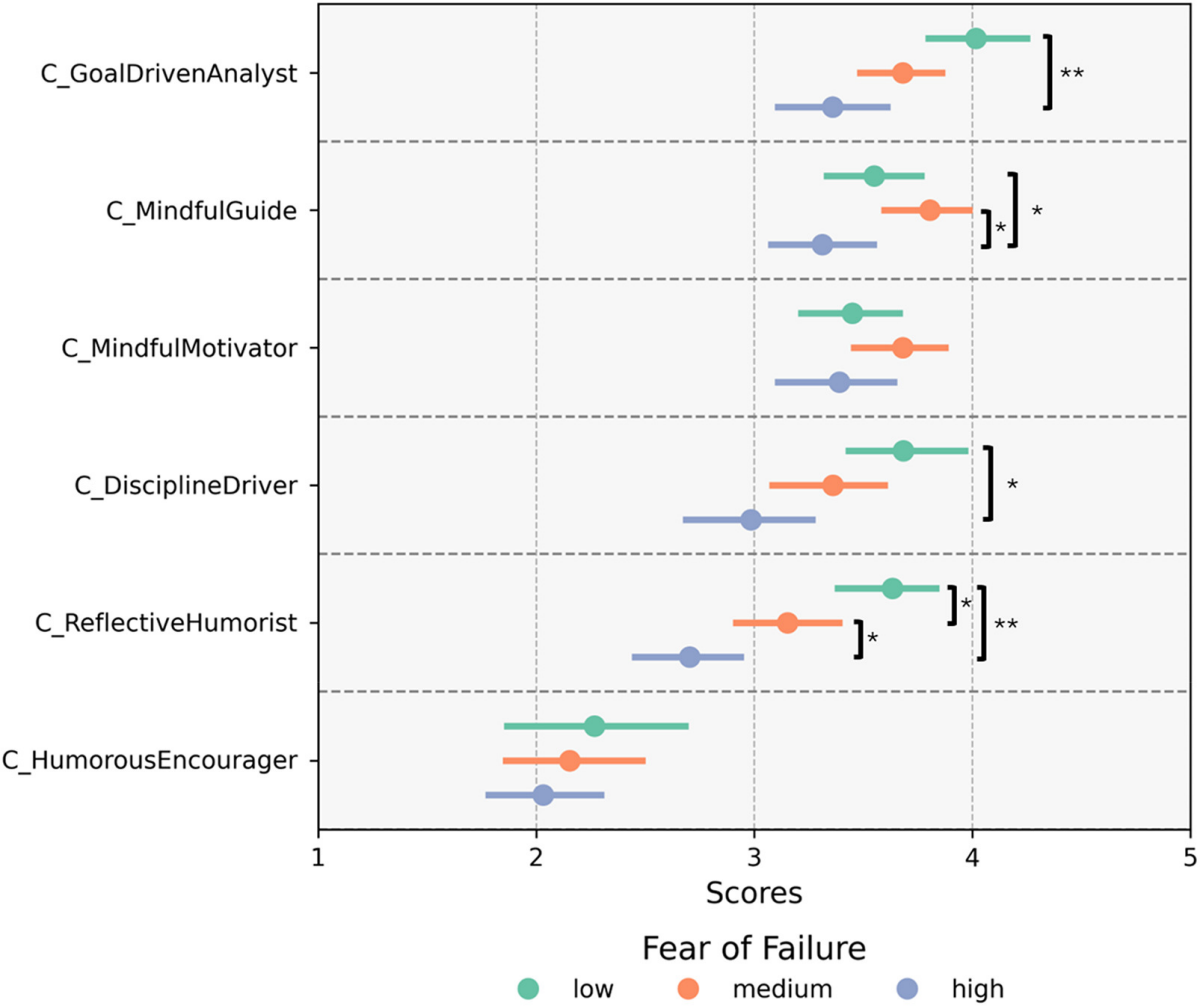
Bootstrap (*n* = 1,000) means 95% confidence intervals on the perceived suitability of the coaches for their training practice separate for each coach and the fear of failure groups (low, medium, high) (1 = completely disagree, 5 = completely agree). *p < 0.05, **p < 0.001. The effect sizes for the significant results range from 0.201 to 0.467 (Cohen's r) and 0.272–0.976 (Cohen's d).

The effect of the exact test for the difference between the coaches inside each group of FoF can be seen [FoF low: *χ*^2^(5, *n* = 60) = 44.8, *p* < 0.001, *W* = 0.149; FoF medium: *χ*^2^(5, *n* = 73) = 87.6, *p* < 0.001, *W* = 0.24; FoF high: *χ*^2^(5, *n* = 65) = 61.8, *p* < 0.001, *W* = 0.19]. The perceived suitability of coaches differed within each group. Figure 3 visualizes the perceived suitability of the coaches for their own training practice separately for each coach and the FoF groups (low, medium, and high). The corresponding detailed *post hoc* test statistics for the between-subject effects are in the Supplementary (Table S1). *post-hoc* tests revealed several significant differences in the perceived suitability of AI coaches. C_HumorousEncourager consistently showed significantly lower perceived suitability than all other coaches, with *p*-values below 0.001 in nearly all comparisons except for one non-significant pairwise comparison.

#### 3.2.3 Fof effects across each coach

Analysis of FoF group effects across coaches revealed notable differences for C_GoalDrivenAnalyst, C_MindfulGuide, C_DisciplineDriver, and C_ReflectiveHumorist (Table 4).

**Table 4:** Fear of failure (FoF) effects in each coach.

Coach	*χ*2	Df	*p*	Eta^2^
C_MindfulMotivator	2.789	2	0.248	/
C_GoalDrivenAnalyst	14.889	2	<0.001**	0.076
C_HumorousEncourager	0.019	2	0.999	/
C_DisciplineDriver	11.515	2	0.003*	0.059
C_ReflectiveHumorist	27.295	2	<0.001**	0.139
C_MindfulGuide	8.153	2	0.017*	0.041

* *p* < 0.05.

** *p* < 0.001.

*Post-hoc* tests are visually displayed in Figure 3; for each of the coaches, C_GoalDrivenAnalyst, C_MindfulGuide, C_DisciplineDriver, and C_ReflectiveHumorist, the groups with high and low FoF significantly differed in their perceptions of the coach's suitability. Persons with a low FoF see themselves as better suited for their training than those with a high FoF.

No additional differences were observed between the moderate FoF group and other groups for C_GoalDrivenAnalyst and C_DisciplineDriver. However, for C_MindfulGuide and C_ReflectiveHumorist, significant differences emerged between the high- and medium-FoF groups. Specifically, participants with a high FoF rated C_ReflectiveHumorist's suitability lower than those with a medium FoF, whereas interestingly, the reverse was true for C_MindfulGuide. Furthermore, low- and medium-FoF participants differed in C_ReflectiveHumorist, with low-FoF participants providing higher suitability ratings.

For *C_ReflectiveHumorist,* all groups differed significantly from the other groups, where the group with a low FoF showed the highest perceived suitability, and the group with a high FoF showed the least perceived suitability.

## 4 Discussion

(R1) The first research question aimed to identify and explore the key characteristics and attribute dimensions defining AI coaches in the context of training support. The results of exploratory and confirmatory factor analyses revealed a multidimensional structure of AI coaching attributes, highlighting four core factors integral to understanding AI's role in training environments. The factor structure uncovered through EFA identified four main dimensions that collectively explained 79.36% of the variance. Overall, the CFA results strongly supported the factor structure derived from the EFA, validating the robustness and reliability of the model. These findings suggest that the hypothesized model accurately represents the underlying structure of the data. These dimensions emerged as distinct but interrelated factors, each reflecting a specific aspect of how AI coaches support their training. The excellent fit indices, including the Comparative Fit Index (CFI) and Root Mean Square Error of Approximation (RMSEA), affirm the robustness of the model, demonstrating that the dimensions identified are significant and stable across samples. The high internal consistency of these factors, as indicated by Cronbach's alpha values ranging from 0.86 to 0.90, further supports the reliability of the dimensions and their relevance in describing the full scope of AI coaching attributes (58). The positive covariances between the factors also point to the interrelated nature of these dimensions, suggesting that the most effective AI coaches will likely exhibit a blend of all four characteristics rather than focusing on any single attribute in isolation.

Factor 1, *knowledge transfer,* reflects an AI coach's effectiveness in delivering precise, valuable, and structured information to trainees. Items such as *informative | contentless, insightful | confusing,* and *educational | not educational* highlight the importance of an AI coach's ability to provide relevant and meaningful knowledge. This factor emphasizes the coach's role as a reliable source of learning and guidance, essential for fostering understanding and progress in training.

Notably, this is an area in which AI coaches have the potential to surpass human coaches, given their ability to access and process vast amounts of background knowledge. This advantage is particularly relevant because studies suggest that many human coaches rely heavily on their existing biographies and practice contexts rather than formal coach education (59). Furthermore, although many coaches recognize the importance of sports science, they do not necessarily incorporate it into their coaching practices (60). By contrast, AI chatbots can draw from a broader range of up-to-date information. However, possessing extensive knowledge alone does not guarantee effective communication. AI chatbots may struggle to convey information engaging or contextually appropriately. One of them is C_HumorousEncourager, which is, in the present study, a domain where human coaches often excel because of their interpersonal skills and adaptability.

Factor 2 emphasizes *goal-oriented persistence*, reflecting an AI coach's ability to encourage perseverance and maintain focus, which is critical for helping trainees achieve their objectives. Attributes such as *persistent | yielding*, and *goal-oriented | aimless* highlight the importance of steadfastness and a clear sense of direction in the coaching process. This factor aligns with research emphasizing the centrality of goal setting and persistence in fostering intrinsic motivation and long-term engagement in training contexts (61–63).

Factor 3, *appreciation and recognition*, captures the AI coach's ability to provide positive reinforcement and acknowledge trainees' efforts. Attributes such as *appreciative* | *disregarding* and *rewarding* | *punishing* reflect the need for AI coaches to build a supportive environment in which trainees feel valued. This aligns with the existing literature, emphasizing the importance of autonomy-supportive behaviors such as acknowledging athletes' efforts, fostering a sense of competence, building trust, and promoting intrinsic motivation in the coach-athlete relationship (27). However, the effectiveness of AI-driven recognition depends on the ability to deliver praise in a genuine and contextually appropriate manner. Although the AI coaches in this study (except for C_HumorousEncourager) received relatively favorable ratings, human coaches may still have an advantage in personalizing recognition. Human coaches can leverage their interpersonal skills to tailor feedback to individual needs and build more profound and meaningful connections, something that AI systems often struggle to replicate, as demonstrated in previous research [e.g., (64)].

Factor 4, *motivational support*, underscores the energizing role of AI coaches. The strong loadings for items like *energizing* | *exhausting* and *motivating* | *demotivating* emphasize the AI coach's capacity to keep trainees engaged and energized. This dimension is central to the idea that AI coaching provides instructions or feedback and maintains the trainee's enthusiasm and drive (24), particularly in long-term training contexts where motivation may fluctuate, as research shows (65).

Overall, these findings underscore the multidimensional nature of AI coaching, emphasizing functional aspects, such as goal setting and determination, and relational dimensions, such as appreciation and motivation. This holistic perspective aligns with contemporary approaches in coaching psychology, which emphasize integrating task-oriented strategies and interpersonal dynamics to foster athlete development, enhance engagement, and promote coaching effectiveness (66, 67). This approach calls for developing emotionally intelligent AI systems in sports, underscoring the importance of creating systems that can adapt and respond to users' emotional and motivational states.

(R2) The second research question investigated whether coaches with varying ratings regarding their suitability for the participants' training practices differed in the associated attributes. The results revealed a relationship between suitability and the attributes linked to different AI coaches. Coaches with higher suitability ratings, such as C_GoalDrivenAnalyst, are associated with qualities that seem to be positively viewed in the coaching context, such as being informative, persistent, appreciative, and energizing. In contrast, C_HumorousEncourager, rated as the least suitable, is more strongly associated with attributes that are consequently perceived as less favorable, including demotivating, less supportive, and disengaging. These findings align with prior AI research in which positive user perceptions were linked to higher satisfaction, engagement, and continued use of AI tools (68–70).

(R3) The third research question analyzed whether AMS-Sport influences the perception of AI coaches' suitability. These findings underscore the critical role of individual psychological traits in the perception of the suitability of different AI coaches. Significant differences in perceptions were found across FoF groups. For specific coaches, the FoF plays a decisive role in determining how suitable individuals perceive the coach for training. Most present studies coach participants with a high FoF and generally perceive them as less suitable for their training practice. By contrast, participants with a low FoF consistently rated most AI coaches as more suitable for training than participants with a high FoF, suggesting that individuals with less performance-related anxiety may find AI coaches more aligned with their needs. Such disparities suggest that those with a high FoF may require additional motivational or emotional support that existing AI coach designs do not adequately address. Previous research on AMS-Sports supports the role of FoF in influencing training perceptions and performance outcomes (71, 72), suggesting that a high FoF can hinder effective engagement and perceived competence in various contexts. By contrast, HfS was not a significant factor in this study, indicating that positive motivational traits may play a lesser role in shaping the perceptions of AI coaches.

The results raise important ethical and practical concerns. While AI coaching systems strive to democratize access, they may inadvertently marginalize users with high FoF by overlooking complex emotional needs—alongside other relevant factors not captured within the scope of this study. This reflects a broader tension in neoliberal frameworks that prioritize efficiency, self-optimization, and scalability over relational, human-centered support (73). Without intentional design, AI risks reinforcing inequities—favoring confident users while underserving those needing deeper emotional engagement. Mitigating this issue involves integrating adaptive personalization mechanisms that leverage real-time user data to harmonize practical guidance and socioemotional assistance, following the approach demonstrated in AI-driven healthcare platforms (74). Moreover, algorithmic bias—already evident in domains like AI hiring (75)—can also affect fitness and training recommendations, reinforcing exclusionary dynamics. Participatory, culturally responsive design frameworks are essential to ensure AI coaches reflect diverse needs and training paradigms (76). C_HumorousEncourager was consistently rated as the least suitable across all FoF groups. The consistently low suitability ratings highlights ethical risks in deploying humor within AI coaching, as its tone risks undermining user trust or perceived competence—a concern amplified by studies showing that poorly contextualized humor can erode authority or alienate users (77). This underscores the need for ethical frameworks, such as the IEEE's *Ethically Aligned Design* principles, to guide AI systems in balancing levity with professionalism, ensuring interactions remain empowering rather than inadvertently demeaning (78). Consequently, there is also the risk that when an AI coach fails to meet individual needs, users may disengage more quickly or abandon the program altogether. This suggests the need to re-evaluate C_HumorousEncourager's approach, highlighting potential areas for improvement in both the AI coach's design and its ability to engage users effectively. This study had several limitations to consider in light of these findings. Implementing AI coaching necessitates careful consideration of safety and ethical concerns. Notably, the study did not address critical safety risks such as AI-generated training recommendations that exceed users' physical limits or propagate incorrect techniques, which could lead to injury or long-term harm. Furthermore, while data privacy vulnerabilities and algorithmic biases (e.g., underrepresenting diverse athlete populations) are acknowledged, their systemic implications for equitable AI adoption in sports coaching remain underexplored, representing a key gap in translating research to real-world applications (79).

The research primarily focused on perceived suitability as an outcome measure without examining how these perceptions translate into tangible behavioral changes, such as improved training adherence, skill acquisition, or performance outcomes. In addressing this gap, future studies should adopt longitudinal designs to assess the direct effect of AI coaching on training efficacy and real-world performance improvements.

Although FoF emerged as a significant factor influencing perceptions, this study did not explore the full spectrum of psychological traits, personality types, or motivational orientations. Additionally, contextual factors such as the type of training, individual experience levels, or cultural influences were not considered. Incorporating these variables into future research could offer a more comprehensive understanding of user needs and preferences.

A further limitation of this study is the potential overlap in attributes across AI coaches, which may obscure meaningful distinctions between their designed personas. While efforts were made to differentiate coaches through unique traits (e.g., humor vs. goal-driven approaches), the observed similarities in user perceptions suggest that further refinement is needed to ensure clearer, functionally distinct profiles in future iterations. Although the study identified the key attribute dimensions of AI coaches, it did not thoroughly examine why specific coaches, such as C_HumorousEncourager, were consistently rated poorly across all groups. Future research should investigate the design and interaction shortcomings of underperforming AI coaches to identify opportunities for improvement and refinement. The developed semantic differential could play a crucial role in this process by guiding the creation of AI coaches based on the identified attribute dimensions, leading to more distinct AI coach profiles. This approach addresses the key observation of the present study that many AI coaches are relatively similar in their associated attributes.

It is also important to note that this study was conducted with German participants. The attributes associated with suitable or unsuitable coaching qualities may be influenced by social desirability biases (80) that vary by country. Therefore, country-specific differences may exist, and the results may not directly apply to other social or cultural contexts. Moreover, the sample in this study exhibited a relatively high level of weekly physical activity, averaging over seven hours per week. Therefore, the findings may differ when applied to lower activity-level populations.

Finally, this study examined pre-extracted chat logs rather than real-time interactions with AI coaches. While this controlled approach enhances comparability and isolates key perceptual factors, it does not capture the dynamic nature of AI coaching, where adaptive responses and follow-up questions could influence user experience. The reliance on static logs, combined with the use of only six predefined coach variations and a specific chatbot context, necessitates careful interpretation of the results, as these constraints may limit the transferability of findings to more diverse or adaptive AI coaching systems. For instance, functionalities like personalized training prescriptions or real-time feedback—which may affect long-term coach suitability—were not exemplified. However, the static evaluation provided valuable insights into users' initial perceptions and motivational responses, which remain foundational for future research. Subsequent studies should explore real-time interactions and adaptive AI behaviors to assess their added practical value in training contexts.

## 5 Conclusion

This study advances understanding of AI coaching by identifying four key factors (knowledge transfer, goal-oriented persistence, appreciation and recognition, as well as motivational support) that shape trainee experiences. The findings highlight the critical need to align AI coach designs with users' psychological profiles—particularly their achievement motives, as evidenced by FoF significant influence on suitability perceptions. Theoretically, the study contributes to HCI frameworks by operationalizing AI coaching attributes and extending the self-determination theory by revealing AI's limitations in fulfilling relatedness needs compared to human coaches. While AI coaches excel in scalable knowledge delivery, their current inability to address complex emotional needs—particularly for high-FoF users—confirms they serve best as complements to human coaches. This is consistent with research in other fields, where an AI-human coach combination leveraging hard data and soft interpersonal skills has proven the most effective (81). A potential model could integrate AI for data-driven tasks while reserving human coaches for emotional support. For practical implementation, ethical safeguards must be prioritized to mitigate algorithmic biases and resist neoliberal tendencies that prioritize efficiency over quality. By systematically addressing these challenges, AI coaching systems will progress toward supporting, or even enhancing, human-centric learning and development.

## Data Availability

The raw data supporting the conclusions of this article will be made available by the authors, without undue reservation.
