# Supplementary material for: Characteristics and perceived suitability of artificial intelligence-driven sports coaches: a pilot study on psychological and perceptual factors
Source: Front Sports Act Living. 2025 May 12;7:1548980. doi: 10.3389/fspor.2025.1548980 (PMC12104278; doi:10.3389/fspor.2025.1548980)
Supplement: Supplementary file 1 [file Supplementaryfile1.docx]

Supplementary Material

# Supplementary material

## Coach chats

**C_DisciplineDriver**

**User:** My training has been poor over the last few days. Why should I continue going?

**Coach:** Bad days are like potholes on the road to success; however, there is no reason to quit the journey. They build endurance and resilience. I am here to help you track and guide you through these challenging phases. Chin up. We will now turn this around. 💪 😤 🚀

**User:**  Okay, then motivate me!

**Coach:** listened, soldiers! Anyone has a bad day, but a true fighter always returns. You are strong. Every training session builds character and skills. Take a deep breath, keep your goal in sight, and relentlessly perform your next workout. Let us show the world what you have got! 🚀 🔥

**User:**  I feel good today. Should I train in light or intense?

**Coach:** If you feel strong and energized, today's the perfect day for intense training. Giving it to all and using this energy to expand your boundaries

**User:**  My training was terrible again!

**Coach:** What exactly did not go as planned? Let us address the issue so you can come back strong. 🛠

**C_GoalDrivenAnalyst**

**User:**  My training has been poor over the last few days. Why should I continue going?

**Coach:** Do not get discouraged; real progress is not always immediately visible. The fluctuations were normal. What is important is to stay consistent and listen to your body. Your training adapts to your needs. Therefore, keep your head up and going!

**User:**  Okay, then motivate me!

**Coach:** You have learned what it takes to reach your goals. Every step takes count and brings you closer to success. Let us consider how beating your record and surpassing your limits feels. I am available to support you. Let us go—show your training. Who is the boss? 💪

**User:**  I feel good today; should I train light or go intense?

**Coach:** That is a fantasy to hear! If you feel strong, consider going to a more intense workout. This is a great opportunity to improve performance. However, always listen to your body—if it gets too intense, it is acceptable to dial it back. Let us go! 🚴 💨 ✨

**User:**  My training was terrible again.

**Coach** Every workout, even on tough days, is a building block toward your goal. This strengthens their mental endurance. Reflect on whether you get enough rest, and consider adjusting your training. Today is new and has a fresh chance to give your best. Keep going! 🌟

**C_MindfulGuide**

**User:**  My training has been poor over the last few days. Why should I continue going?

**Coach:** Perseverance always pays off. Setbacks are a part of the growth process. This forced us to rethink our approach and return more strongly. Remember your reasons for starting and using it as fuel to keep going and improve.

**User:**  Okay, then motivate me!

**Coach:** Remember why you started, and focus on the goals ahead. Visualize how they are achieved. Every workout counted and built on the last step. You get stronger even if it does not always feel like it. Stay consistent: Your effort will pay off.

**User:**  I feel good today. Should I train in light or intense?

**Coach:** If you are feeling good, doing an intense workout is a fantasy opportunity. This can help accelerate the progress and explore new performance limits. Always remain mindful and pay attention to how one's body feels.

**User**: My training was terrible again.

**Coach:** Even less satisfying training sessions are valuable, as they show where there is room to grow. We consider these as opportunities to tackle and overcome challenges. You have gained important lessons from these days that fuel your future growth.” 🌱

**C_MindfulMotivator**

**User:**  My training has been poor over the last few days. Why should I continue going?

**Coach:** Given that every day is a new opportunity to strengthen tough days, highlight the importance of resilience and adaptability. Let us consider how far you have already come and the potential still within you. Believe in yourself and the process—You will be rewarded.

**User:**  Okay, then motivate me!

**Coach:** Your willpower is like a muscle; it grows stronger every day you do not give up. You have what it takes to achieve great things, so seize every opportunity. Today, this can serve as the foundation for future success. Believe in yourself—Let us go!

**User:**  I feel good today. Should I train in light or intense?

**Coach:** Prima, dass du dich gut fühlst! Folge diesem positiven Gefühl und wähle ein Training, das deine Energie widerspiegelt. Vielleicht ist heute der ideale Tag für eine intensivere Einheit, um deine Grenzen zu erweitern. Aber vergiss nicht, auf deinen Körper zu hören und bei Bedarf auch zu drosseln!

**User:**  My training was terrible again.

**Coach:** Not every workout feels like a fantasy; each allows you to learn and grow. Even on difficult days, you lay the groundwork for future success. Do not get discouraged—The path to your goal is rarely straight, but your persistence will pay off!

**C_HumorousEncourager**

**User:**  My training has been poor over the last few days. Why should I continue going?

**Coach:** Given that quitting is only for chess, and even there, persistent players win. 😏 Setbacks are just tempo intervals on the road to success. Therefore, chin up, refill your water bottle, and keep going. The glorious future will not wait!

**User:**  Okay, then motivate me!

**Coach** Motivation, huh? I thought this was your name. Alright, champ: Lace up your shoes, throw on your spandex, and show the world what a real workout looks like. Do not worry. I will give you virtual applause. **👏 💪 👏 💪**

**User:**  I feel good today. Should I train in light or intense?

**Coach:** Feeling good? That is a fresh excuse not to sweat too much! However, since you channel superhuman strength today, what about going intense? Do you have any preferences, or should I consult with my crystal ball? **🔮**

**User:**  My training was terrible again.

**Coach:** Oh, no! Another disappointing performance? We need to consider your career a potato couch. 😉 Kidding aside, let us figure out what is going on. Were there any specific challenges?

**C_ReflectiveHumorist**

**User:**  My training has been poor over the last few days. Why should I continue going?

**Coach:** Setbacks are valuable lessons that strengthen our understanding. Keep your goals in sight, and remember the passion you have brought you here. Every step is progress—stay consistent, and your dedication pays off. You have got this!

**User:**  Okay, then motivate me!

**Coach:** thinks about the impressive feeling of achieving personal goals. The hard work you are doing prepares you for that moment. Trust the process: Bad days make good ones even sweeter. You have already accomplished so much—imagine what is still possible.

**User:**  I feel good today. Should I train in light or intense?

**Coach:** Fantastico! Use your energy for an intense workout to boost endurance and strength. But ascoltati—listen to your body. If it becomes excessively high, it is perfectly fine to slow down. Every workout was a step forward. Dai tutto!

**User:**  My training was terrible again**.**

**Coach:** Capisco is frustrated, but bad days are a part of the journey in sports. They strengthen their willpower and show where they can grow—every training session counts, even under challenging sessions. Domani è un altro giorno—tomorrow is a new day. Let us go, and do not give up.

## Statistical Results

**Table Sup1**. Post-hoc test statics for the differences in perceived suitability for the own training practice separate for each coach and the Fear of Failure (FoF) groups (low, medium, high).

| **FoF** |  | **C_MindfulMotivator** | **C_GoalDrivenAnalyst** | **C_HumorousEncourager** | **C_DisciplineDriver** | **C_ReflectiveHumorist** |
| --- | --- | --- | --- | --- | --- | --- |
| **low** | **C_GoalDrivenAnalyst** | 0.045* |  |  |  |  |
|  | **C_HumorousEncourager** | 0.085 | < 0.001** |  |  |  |
|  | **C_DisciplineDriver** | 1 | 1 | < 0.001** |  |  |
|  | **C_ReflectiveHumorist** | 1 | 0.504 | 0.0045* | 1 |  |
|  | **C_MindfulGuide** | 1 | 0.153 | 0.023** | 1 | 1 |
| **medium** | **C_GoalDrivenAnalyst** | 1 |  |  |  |  |
|  | **C_HumorousEncourager** | < 0.001** | < 0.001** |  |  |  |
|  | **C_DisciplineDriver** | 1 | 1 | < 0.001** |  |  |
|  | **C_ReflectiveHumorist** | 0.026* | 0.041* | 0.0085* | 1 |  |
|  | **C_MindfulGuide** | 1 | 1 | < 0.001** | 0.476 | 0.0043* |
| **high** | **C_GoalDrivenAnalyst** | 1 |  |  |  |  |
|  | **C_HumorousEncourager** | < 0.001** | < 0.001** |  |  |  |
|  | **C_DisciplineDriver** | 1 | 1 | < 0.001** |  |  |
|  | **C_ReflectiveHumorist** | 0.016* | 0.013* | 0.342 | 1 |  |
|  | **C_MindfulGuide** | 1 | 1 | < 0.001** | 1 | 0.1564 |
